# Measuring Coverage in MNCH: Challenges and Opportunities in the Selection of Coverage Indicators for Global Monitoring

**DOI:** 10.1371/journal.pmed.1001416

**Published:** 2013-05-07

**Authors:** Jennifer Harris Requejo, Holly Newby, Jennifer Bryce

**Affiliations:** 1 Institute for International Programs, Department of International Health, The Johns Hopkins University, Baltimore, Maryland, United States of America; 2 Statistics and Monitoring Section, Division of Policy and Strategy, UNICEF, New York, New York, United States of America; Professor of Demography and Social Statistics, University of Southampton, United Kingdom

## Abstract

Global monitoring of intervention coverage is a cornerstone of international efforts to improve reproductive, maternal, newborn, and child health. In this review, we examine the process and implications of selecting a core set of coverage indicators for global monitoring, using as examples the processes used by the Countdown to 2015 for Maternal, Newborn and Child Survival and the Commission on Accountability for Women's and Children's Health. We describe how the generation of data for global monitoring involves five iterative steps: development of standard indicator definitions and measurement approaches to ensure comparability across countries; collection of high-quality data at the country level; compilation of country data at the global level; organization of global databases; and rounds of data quality checking. Regular and rigorous technical review processes that involve high-level decision makers and experts familiar with indicator measurement are needed to maximize uptake and to ensure that indicators used for global monitoring are selected on the basis of available evidence of intervention effectiveness, feasibility of measurement, and data availability as well as programmatic relevance. Experience from recent initiatives illustrates the challenges of striking this balance as well as strategies for reducing the tensions inherent in the indicator selection process. We conclude that more attention and continued investment need to be directed to global monitoring, to support both the process of global database development and the selection of sets of coverage indicators to promote accountability. The stakes are high, because these indicators can drive policy and program development at the country and global level, and ultimately impact the health of women and children and the communities where they live.

## Abstract 2

In a *PLOS Medicine* Review, Jennifer Requejo, Holly Newby and Jennifer Bryce discuss the five-step process that underlies the generation of data for global monitoring of intervention coverage for maternal and child health and describe the problems associated with selecting appropriate coverage indicators for global monitoring.

*This paper is part of the* PLOS Medicine “*Measuring Coverage in MNCH" Collection*.

## Introduction

Global monitoring of coverage for maternal and child health interventions involves the collection and analysis of a limited set of quantitative indicators to assess progress, and is central to international efforts to improve reproductive, maternal, newborn, and child health (RMNCH). Decision makers use results from global monitoring to set priorities and to determine where to allocate resources [1]. Coverage measures are a major focus of global monitoring because they can change much more rapidly in response to policy and program interventions than measures of impact (e.g., mortality, morbidity, fertility, nutritional status). Coverage refers to the proportion of a population in need of a public health intervention that actually receives it [2].

In this article, which is part of the *PLOS Medicine* “Measuring Coverage in MNCH" Collection, we review the steps involved in producing data of adequate quality for use in global monitoring. We also provide a critical analysis of the sets of coverage indicators included in the Countdown to 2015 for Maternal, Newborn and Child Survival and the Commission on Information and Accountability for Women's and Children's Health initiatives (Table 1), which are referred to as “Countdown" and “the Commission," respectively, throughout the rest of this review. Because global monitoring results affect the lives of women and children, it is critical that the “right" indicators are assessed and correctly interpreted. Thus, our aim in this article is to recommend improvements in the process used to select sets of coverage indicators in global monitoring efforts moving forward to and beyond 2015.

**Table 1:** Countdown to 2015 for Maternal, Newborn and Child Survival and the Commission on Information and Accountability for Women's and Children's Health.

*Countdown to 2015 for Maternal, Newborn and Child Survival*	*Commission on Information and Accountability for Women's and Children's Health*
**Aim**	**Aim**
Focuses on coverage and uses country-specific data to stimulate and support country progress towards the health related MDGs, particularly MDG 4 and MDG 5.	To develop a framework for global reporting, oversight and accountability on women's and children's health.
**Organizational structure**	**Organizational structure**
A global movement of academics, governments, representatives of multilateral and bilateral agencies, professional associations, non-governmental organizations and other members of civil society. It has a governance structure that manages the work and inputs from over 70 members.	Time-limited group developed following the launch of the Global Strategy for Women's and Children's Health in 2010. Progress in implementing its recommendations is overseen by an independent Expert Review Group (iERG).
**Countries**	**Countries**
The 75 countries where more than 95% of all maternal and child deaths occur.	The 75 countries where more than 95% of all maternal and child deaths occur.
**Products, reporting, and dissemination**	**Reporting and dissemination**
Periodic reports (in 2005, 2008, 2010 and 2012) and country profiles on key aspects of reproductive, maternal, newborn and child health. Advocacy materials and peer-reviewed articles.	In May 2011, the Commission launched its report, *Keeping Promises, Measuring Results*. The iERG will report annually until 2015 on progress in implementing its 10 recommendations. The first report was published in September, 2012.

## The Process of Global Monitoring

**Figure 1:**
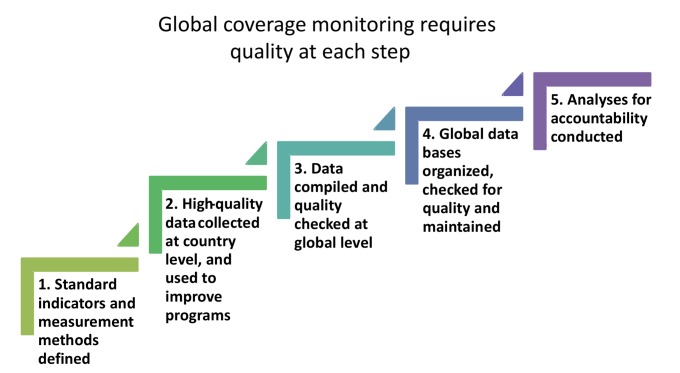
The five-step process for global monitoring of intervention coverage.

Five iterative steps are required to generate and use coverage data for global monitoring (Figure 1).

First, global consensus indicators must be defined. The characteristics of a “good" indicator for global monitoring include: high validity (the extent to which the indicator is a true and accurate measure of the phenomenon under study); reliability (the extent to which indicator measurements are consistent and dependable across countries and over time); the ability to detect change within a reasonable period and as a result of program implementation; and the ability to produce data that are easily interpreted and therefore useful in guiding program change [3],[4].

Second, each indicator must be measured at the country level using standard methods that produce complete, comparable, high-quality data that are nationally representative. Producing such data requires specialized technical inputs in sampling and survey design, thorough training and supervision of those who collect the data, and close attention to quality in data entry, cleaning, weighting, and tabulation. Another paper in this Collection reviews the Demographic and Health Surveys and the Multiple Indicator Cluster Surveys, the two international household survey programs that produce the majority of coverage data used in global monitoring [5].

Third, country-level data must be compiled at the global level. This step involves checking data quality and the consistency of indicator measurement and often requires the recalculation of indicators from raw datasets. This process is labor intensive and contributes to the time lag between completion of surveys and the public availability of compiled databases. Various agencies (including UNICEF, WHO and UNFPA, and Save the Children) lead this step for different indicators.

The fourth step entails organizing the global databases and conducting another round of data quality checking. For some indicators (e.g., immunization coverage), this step includes a consultative process that involves the United Nations and independent technical groups, who develop estimates based on the combination of survey and program data. Country consultations are held on these adjusted indicators prior to their inclusion in publicly accessible databases.

Alteration of global databases to incorporate new interventions and improved measurement approaches requires adjustments in steps one through four. Decisions to introduce changes to global databases need to be made through a consultative process so that reporting requirements remain feasible and relevant for countries, trend analyses remain possible, and consensus is reached on indicator importance for informing programs and policies.

The fifth and final step is the completion of specific accountability analyses. This step requires agreement on which countries will be included, which indicators will be highlighted, what additional information is needed to interpret the results, and the identification of key messages for target audiences. These analyses must take into account any limitations of the data that are identified in earlier steps to ensure that correct interpretations are made for public health programming.

## Defining Sets of Indicators for Global Monitoring: Countdown and the Commission

Over the years, several initiatives, including Health for All by the Year 2000 [6], the World Summit for Children [7], and the Millennium Development Goals (MDGs) [8],[9], have monitored sets of indicators to assess global progress in health. Critiques of these initiatives (e.g., [10],[11]) stress that sets of global indicators represent more than the sum of the individual measurements, and often reflect broader aspirational concepts such as human development and human rights. The eight MDGs, for example, have been considered as a stimulus to poverty reduction strategies [12], official development assistance and political consensus [13], and increased monitoring of development projects [10]. Others argue that the definition of the MDGs is too narrow and leaves some important areas associated with development unrepresented, that the synergies across the MDGs are not sufficiently clear, and that they are particularly silent on equity [11].

The Countdown and Commission initiatives include sets of indicators that monitor MDG 4 (reduce child mortality) and MDG 5 (improve maternal health). Both initiatives address some of the limitations of the MDG framework, at least for the health of women and children, by embracing the holistic concept of the continuum of care and stressing the interrelatedness of the two MDGs. Both also complement the MDG indicators with recommendations that a more expansive set of RMNCH coverage indicators are analyzed nationally and by key equity considerations to promote accountability. Table 2 shows the coverage indicators in the two initiatives and compares them to the RMNCH coverage indicators included in MDG monitoring. In the next two subsections, we describe the technical and political considerations that drove the selection of the indicator sets tracked by Countdown and the Commission.

**Table 2:** Coverage Indicators for Global Monitoring of RMNCH: Millennium Development Goal Framework, Countdown to 2015 for Maternal, Newborn and Child Survival, and the Commission for Information and Accountability for Women's and Children's Health, 2012.

Coverage Indicator		Millennium Development Goal^a^	Countdown to 2015	Commission	Issues in Indicator Comparability across Initiatives
***Pre-pregnancy***					
1)	Demand for family planning satisfied		X	X	
2)	Contraceptive prevalence rate	X			Countdown includes this indicator in a supplemental webannex to its report
3)	Unmet need for family planning	X			Countdown includes this indicator in a supplemental webannex to its report
***Pregnancy***					
4)	Antenatal care (at least one visit) with a skilled provider	X	X		
5)	Antenatal care (four or more visits) by any provider, skilled or unskilled	X	X	X	Commission indicator is defined as skilled provider only. Data are not currently available through international household survey programs for skilled provider.
6)	Intermittent preventive treatment of malaria for pregnant women		X		
7)	Neonatal tetanus protection		X		
8)	Prevention of mother-to-child transmission of HIV		X	X	The Commission combines the two HIV indicators; MDG 6B called for the achievement, by 2010, of universal access to treatment for HIV/AIDS for all those who need it. Target indicators for prevention of mother-to-child-transmission of HIV or antiretrovirals for pregnant women are not listed in the MDG framework.
9)	Eligible HIV+ pregnant women receiving anti-retroviral therapy for their own health		X	X	
***Birth***					
10)	Skilled attendant at birth	X	X	X	
11)	Cesarean section rate		X		
***Postnatal***					
12)	Early initiation of breastfeeding		X		
13)	Postnatal visit for mother		X	X	The Commission reports on the two postnatal care indicators as a composite measure. Data may be available through international household survey programs on the composite measure in the current and future survey rounds.
14)	Postnatal visit for baby		X	X	
***Infancy***					
15)	Exclusive breastfeeding		X	X	
16)	Introduction of solid, semi-solid, or soft foods		X		
17)	Diphtheria-tetanus-pertussis (three doses)		X	X	
18)	Measles immunization	X	X		
19)	*Haemophilus influenzae* type b immunization (three doses)		X		
20)	Vitamin A supplementation (two doses)		X		
***Childhood (under the age of 5 years)***					
21)	Children sleeping under insecticide-treated nets	X	X		
22)	Children with fever receiving first line antimalarial treatment	X	X		
23)	Careseeking for pneumonia		X		
24)	Children with suspected pneumonia receiving antibiotic treatment		X	X	
25)	Oral rehydration therapy with continued feeding		X		
26)	Oral rehydration salts		X		
27)	Improved drinking water sources	X	X		
28)	Improved sanitation facilities	X	X		

a includes only MDG target indicators related to RMNCH.

### Selection of Indicator Sets by Countdown

Since its inception in 2003 by the Bellagio Study Group on Child Survival [14], Countdown (Table 1) has produced periodic reports and country profiles on key aspects of RMNCH, and has been widely recognized for its role in promoting the use of coverage data to hold stakeholders to account for global and national action.

Countdown tracks progress in the 75 countries where more than 95% of all maternal and child deaths occur [15]. It synthesizes data on coverage of lifesaving interventions across the continuum of care, highlighting successes and missed opportunities. Countdown also tracks key determinants of coverage—equity patterns across population groups, health system factors, supportive policies, and available financial resources—and takes into consideration the role of broader contextual factors in driving coverage change [16].

Countdown is not involved in steps one through four in the global monitoring process (Figure 1). These steps are implemented by UNICEF, in consultation with various technical groups, resulting in a public access database (childinfo.org) that is updated annually and that contains the most recent estimates for coverage indicators for all countries. Countdown abstracts coverage data from this database and conducts a further check on internal and external validity by looking for out-of-range values or inconsistencies with other national survey data. Countdown then presents these data on country profiles and carries out secondary analyses to produce reports. This last stage includes a series of consultations with the Countdown membership to agree on what the coverage results mean for global public health, what key messages can be drawn to spur action, and how (and to whom) those messages can best be communicated.

Countdown has defined criteria to guide the selection of interventions for which it will track coverage. The most important criterion is the availability of internationally accepted evidence demonstrating intervention effectiveness in reducing maternal, newborn or child mortality and feasibility for delivery at scale in low- and middle-income countries. In addition, each intervention tracked by Countdown must be associated with a “good" coverage indicator as defined earlier. Countdown has reviewed its coverage indicators three times. The review process includes an assessment of experience in the last reporting cycle, consideration of new interventions and their associated coverage indicators against the selection criteria, and an open solicitation of proposals for changes.

Countdown reports on the number of countries with recent data for each indicator that it tracks. In its 2012 report, this ranged from only four countries with data for postnatal care for the baby to 73 countries with comparable data on measles [17]. The report also showed that only 29 Countdown countries had conducted a household survey during 2009–2011. These findings are presented to emphasize the need for more data collection efforts in the 75 countries as a prerequisite to improved accountability.

### Selection of Indicator Sets by the Commission

In 2010, a global strategy led by the United Nations called “Every Woman, Every Child" gave rise to a time-limited Commission on Information and Accountability for Women's and Children's Health. The Commission's mandate was to develop a framework for global reporting, oversight and accountability on women's and children's health [18].

The Commission used a two-step process to select a set of 11 core indicators, including three impact and eight coverage measures. Seven of the core coverage indicators are measured primarily through household surveys, and are described in Table S1 according to the criteria for what makes a “good" coverage indicator. National program records aggregated from facility records and modeling techniques are used to generate estimates for the remaining core coverage indicator—prevention of mother-to-child transmission of HIV and antiretroviral therapy for pregnant women. To select its core indicators, the Commission first convened a Working Group on Accountability for Results to prepare a background paper on recommendations for a set of indicators and measurement needs for women's and children's health. The background paper [19] specified that the core set of indicators should be limited in number to reduce the reporting burden on countries, should be reflective of the continuum of care, and should have strong political and public health significance across countries. The Working Group reviewed the MDG and Countdown indicators and recommended a set of coverage indicators from these. The Commission's final report – *Keeping Promises, Measuring Results* – incorporates these recommendations [18]. Other papers in this Collection examine the performance of several of these indicators with the aim of improving their measurement and interpretation [20]–[24]. Countdown, in collaboration with the Health Metrics Network, has also produced a report that describes the program relevance and measurement limitations of the coverage indicators selected by the Commission [25].

The Commission report also called for the creation of an independent Expert Review Group (iERG) to report annually until 2015 on progress in implementation of the Commission's 10 recommendations, which include improved measurement of the core coverage indicators in the 75 countries. Their first report indicated that, in 2012, only 11 of the 75 countries had recent data for all eight core coverage indicators, that there were gaps in coverage along the continuum of care, and that the poorest groups disproportionately experienced the lowest levels of coverage. The iERG recommended expanded commitment and capacity to evaluate RMNCH initiatives in order to help countries set priorities and allocate resources accordingly [26].

## Tensions and Compromises in the Selection of Global Monitoring Indicators

The selection of core coverage indicators by Countdown and by the Commission illustrates four tensions inherent in the process of selecting indicator sets for global coverage monitoring.

A first tension is between the desire to have ***comprehensive information*** about the policies and programs relevant to the topic of interest (i.e., poverty reduction, RMNCH), and the simultaneous need to ***keep the number of indicators small*** to minimize the reporting burden on countries and to avoid information overload. Resolution of this tension entails making hard choices about which indicators are left out. Indicators by definition are signals of the need to investigate a phenomenon more thoroughly. That this concept is poorly understood is reflected by the importance often mistakenly accorded to individual indicators in decision-making processes. Those engaged in global monitoring must continue to educate their target audiences about the appropriate use of indicators as signals that can trigger the need for further investigation and as signals that should be interpreted in the context of more comprehensive information.

Both Countdown and the Commission faced the challenge of selecting a core set of coverage indicators that represent the continuum of care as well as a balance between preventive and curative interventions. One way in which both initiatives achieved this was by selecting indicators of service contacts that reflect major dimensions of the continuum (e.g., antenatal care for the pregnancy period, skilled attendant at birth for labor and delivery, and postnatal care visits for the postnatal period). Although measurable through household surveys and readily understood, these contact measures do not necessarily reflect receipt of recommended interventions, which limits their usefulness for programmatic purposes.

A second strategy used by Countdown, but not by the Commission, to address this first tension was to expand the number of core coverage indicators, and to track coverage determinants (e.g., health systems factors, policies, and financial data). Starting in 2008, Countdown extended its country profiles to two pages and created web-based supplemental tables to provide additional information. Countdown aims to be responsive to new evidence and regularly reviews its indicator set to ensure that it captures the best information available on RMNCH coverage. This iterative approach can, however, result in the selection of too many indicators and a consequent loss of focus and an inability to generate well-targeted key messages. Within the Commission, by contrast, the power to make decisions about the indicator set rested firmly with the seven Commissioners, informed by technical experts through the Working Group. This structure allowed the Commission to keep the total number of indicators small and its effort focused, but may have resulted in critical gaps. The Commission set, for example, includes only one indicator for prevention of childhood illnesses (diphtheria-tetanus-pertussis vaccination) and one indicator for case management of childhood illnesses (antibiotic treatment of childhood pneumonia). The set does not address interventions related to malaria or diarrhea, two major causes of child deaths in most of the 75 countries. The Commission acknowledges, therefore, that its indicator set is not sufficient for country-level monitoring.

A second tension is between ***what we know is important*** for improving public health and ***what can be measured reasonably well*** given available data sources and methods. A good example of compromise is the inclusion by both Countdown and the Commission of an indicator on coverage for antibiotic treatment of childhood pneumonia. Pneumonia, the number one killer of children worldwide [27] can be treated effectively and at relatively low cost with a course of antibiotics [28] but, as reported elsewhere in this Collection [20],[21] and summarized in Table S1, the current global coverage indicator for treatment does not produce accurate results. Its measurement limitations suggest that additional related indicators may need to be collected until data collection methods are improved (i.e., care seeking for pneumonia). Countdown takes this approach and tracks both careseeking and treatment indicators. Another example is the Commission's antenatal care indicator (“four or more antenatal care visits from a skilled provider"). Although this indicator could potentially provide more programmatically useful data than the Countdown and MDG target indicator (which specifies any provider), in practice, information on the type of provider for each of four or more visits cannot be measured through household survey interviews because of recall issues. The inclusion of this indicator in the Commission set may spur efforts to collect these data, but comparable data from the 75 countries are currently not available. These examples demonstrate that when there is not a single indicator that is technically sound and useful for guiding programs for a given topic area, global monitoring initiatives can opt to collect a set of indicators related to the topic or provide adequate documentation on how to interpret a less-than-ideal indicator.

A third tension is between focusing on coverage for ***interventions that address the highest disease burden*** and ensuring that the indicator is ***relevant to as many countries as possible***. Two good examples of this tension concern malaria and HIV/AIDS. In two-thirds of the 75 countries covered by Countdown and the Commission, at least 75% of the population is at risk of *Plasmodium falciparum* malaria transmission. Interventions that effectively prevent deaths from malaria, such as the provision and use of insecticide-treated nets, antimalarials, and intermittent preventive treatment for pregnant women, have been scaled up rapidly in many of these countries [17]. For HIV/AIDS, 21 of the Countdown/Commission countries are priority countries for the elimination of mother-to-child transmission of HIV because of high levels of HIV seroprevalence in women. Prevention of mother-to-child-transmission of HIV with antiretroviral therapy drugs has been shown to be effective and is being rapidly scaled up in these countries [29]. Countdown reports on coverage of all these interventions; the Commission only reports on coverage of HIV/AIDS interventions (Table 2). These different choices show that the selection processes for global monitoring are not strictly based on the technical merits of individual indicators.

A fourth tension is between the need for ***timely data to guide decision making*** and the ***cost and resources required to conduct frequent surveys***. Frequent and high-quality coverage data are essential for program monitoring and require regular implementation of household surveys that meet at least minimum quality standards. In their reports, both Countdown and the iERG highlight data gaps for the indicators they track across and within the 75 countries they cover, and make strong arguments for frequent—even annual—collection of coverage data. Although a criterion for indicator inclusion in Countdown is data availability in most of the 75 countries to enable results to inform programs and policies, exceptions have been made for a few indicators because of their public health importance. Coverage for postnatal care for newborns, for example, has been included in Countdown reporting in the last two cycles [17],[30] based on its importance for neonatal survival, even though fewer than 10 countries had data to report. It is now also one part of a composite indicator recommended by the Commission. This move towards reporting postnatal care for newborns has raised the visibility of postnatal care and has flagged the need to increase data collection efforts. Importantly, investments in data collection efforts to address such gaps must always be based on careful consideration of the time-frames required for detecting changes in specific indicators. Table S1 details variations in the ability of the core Commission indicators to detect change over time. Some indicators, such as exclusive breastfeeding and proportion of demand for family planning satisfied, are highly responsive to changes as they reflect current coverage. Others, such as skilled attendant at birth, are based on recall periods of two and five years (for the Multiple Indicator Cluster Surveys and Demographic and Health Surveys, respectively). Building capacity at the country level on understanding variations in the responsiveness of coverage indicators is essential for planning data collection efforts in view of resource constraints and for interpreting coverage levels and trends.

## Conclusions and Recommendations for Global Monitoring

Global monitoring efforts should produce timely results that can be used to support sound policy and programmatic decisions. Efforts are needed to generate better coverage data so that the indicators selected for global monitoring meet all technical requirements and to improve country capacity to measure, report on, and use them. In this article, we have reviewed the steps involved in global monitoring, the processes used by Countdown and the Commission to select a subset of coverage indicators for tracking coverage, and key tensions associated with selecting coverage indicators for global monitoring.

Several lessons gleaned from the Countdown and Commission indicator selection processes can be applied to the work now underway to define goals for the post-2015 era. First, the indicator selection process should be guided by the technical merits of individual indicators as well as by the underlying aspirational goal or broader agenda at the heart of the monitoring effort. Both the Countdown and Commission selection processes were driven in large part by the need to ensure that the indicator set adequately captures information across the continuum of care. Results can be used to identify major gaps and successes along the continuum, and to hold all partners to account for progress in achieving MDG4 and MDG5.

Second, any core set of coverage indicators needs to be interpreted within the context of information on inputs, processes and outputs related to program and policy implementation as well as broader social, economic, political, and environmental information that might affect coverage levels and trends. Countdown routinely tracks determinants of coverage in its analyses, and the Commission is clear on the need for additional information to supplement its coverage results, particularly at the national level.

Third, coverage indicators need to be selected through a rigorous and transparent process that involves consultation with a wide range of stakeholders. The process should include assessment of intervention effectiveness, data availability, and quality. The measurement limitations of the indicators also need to be identified so that they can be taken into consideration when interpreting results.

Our recommendation for the post-2015 agenda-setting process is that the example of the Commission should be followed, but that an additional step that involves critical review of the proposed indicators should be added to ensure that the indicators reflect the best balance between feasibility of measurement, data availability, and the broader agenda-setting functions needed from the set as a whole [13]. Such a review process should also stimulate further investment in a program of measurement research to improve the “value" of coverage indicators. For example, Table S1 shows that the development of methods for capturing information on interventions received during service contacts (e.g., antenatal care, skilled attendant at birth, and postnatal care) that is representative at the population level requires further work. Support for ongoing efforts to ensure consistency of measurement between the major international survey programs and over time is also needed for the production of comparable data.

Initiatives like Countdown with a more expansive indicator set should continue to serve as a resource for higher-level global monitoring efforts and to guide program development and implementation at national and sub-national levels. The bottom line, however, is that effective global monitoring depends on all five steps in the global monitoring process, and it is imperative that the whole process receives sufficient resources to allow it to respond to the future health needs of mothers and children.

**Key Points**

- Tracking coverage of interventions proven to reduce maternal, newborn, and child mortality is central to global monitoring efforts.
- Effective global monitoring depends on a five-step process that ensures the generation of high-quality data.
- Sets of coverage indicators selected for global monitoring play a key role in driving policy and programmatic decisions at the global and national levels, but it is essential that other related information is considered when making these decisions.
- Correct interpretation of levels and trends of coverage indicators depends upon awareness of their strengths and limitations, and on the commitment and ability of stakeholders to use them for decision making.
- Efforts are needed to improve country capacity to measure and report on core coverage indicators through household surveys and routine reports; moreover, a rigorous and inclusive technical review process is needed to ensure that indicators for post-2015 global monitoring are chosen on the basis of evidence of effectiveness, feasibility of regular measurement, and programmatic relevance, and to maximize uptake of the indicators.

## Supporting Information

Table S1 Assessment of the seven core coverage Commission indicators measured primarily through household surveys.
(DOC)

## Abbreviations

**iERG**: independent Expert Review Group
RMNCH: reproductive, maternal, newborn, and child health
MDG: Millennium Development Goals
