# Supplementary material for: Measuring Coverage in MNCH: Challenges and Opportunities in the Selection of Coverage Indicators for Global Monitoring
Source: PLoS Med. 2013 May 7;10(5):e1001416. doi: 10.1371/journal.pmed.1001416 (PMC3646210; doi:10.1371/journal.pmed.1001416)
Supplement: Table S1 — Assessment of the seven core coverage Commission indicators measured primarily through household surveys. (DOC) [file pmed.1001416.s001.doc]

**Table S1.** **Assessment of the seven core coverage Commission indicators measured primarily through household surveys***

| **Coverage indicator** | **Public health importance** | **Validity** | **Reliability** | **Ability to detect change over time** | **Interpretability** |
| --- | --- | --- | --- | --- | --- |
| **Proportion of demand for family planning satisfied** | Enables assessment of family planning programs and progress in providing contraceptive services to women who wish to avoid pregnancy | Accuracy of women’s reporting on use of contraceptives may vary by method and needs further study | May be affected by differences in questions, question sequencing and indicator calculations across different surveys | Should respond rapidly to activities because it measures reported contraceptive use at the time of the survey | Because estimates are based on a complicated algorithm, they may be difficult to interpret by decision makers. Women who are using traditional methods of contraception are considered to have a met need for contraception. Because these methods can be less effective than modern methods, the indicator may not accurately reflect met need. |
| **Antenatal care (four or more visits) with a skilled provider** | WHO recommends four or more visits for uncomplicated pregnancies. Life saving interventions for the mother and baby should be provided during these visits | Women may not know and may not be able to recall the skill level of the provider for every visit received. Current survey protocols only collect general data on type of provider. | The reliability of this antenatal care indicator has not been assessed. MICS and DHS survey protocols use different recall periods (births that occurred two and five years prior to the survey), although the indicator for the two year period can be calculated from DHS data. | Data from the two international survey programs reflects information on pregnancies that occurred two to five years prior to the survey. This reduces the responsiveness of the indicator to immediate programmatic or policy changes on care during pregnancy | The indicator captures information about whether women received antenatal care during pregnancy, but does not provide information about receipt of any recommended interventions. This limits the usefulness of the indicator for programmatic purposes or for assessing service quality. |
| **Skilled attendant at birth** | Serves as a process measure of the health system’s ability to provide adequate care for pregnant women during labour and delivery | Survey questionnaires must be adapted to reflect national health cadres; respondents need to be able to report on who attended the delivery; and the appropriate “skilled” cadres need to be identified and included during data compilation and analysis. Personnel identified as skilled may not actually have received the requisite training or have the needed experience to provide adequate care. | MICS and DHS survey protocols use different recall periods (births that occurred two and five years prior to the survey), although the indicator for the two year period can be calculated from DHS data. And, surveys may differ in the list of cadres included. | Data from the two international survey programs reflects information on pregnancies that occurred two to five years prior to the survey. This reduces the responsiveness of the indicator to immediate programmatic or policy changes on care during labour and delivery | Captures information only on whether a woman received care from a skilled provider, but not on whether effective interventions with proven benefits for maternal and perinatal health were received. Information on availability of needed supplies and equipment is also not captured. This limits the usefulness of the indicator for informing programs and policies. |
| **Postnatal care for mothers and babies** | The majority of maternal and newborn deaths occur within the first 48 hours after birth. Postnatal care, especially within the first few days, are an opportunity to provide effective interventions to improve maternal and newborn health | DHS and MICS capture information on PNC separately for mothers and babies. Technical work is needed to assess the feasibility and validity of global reporting on a combined indicator. MICS tables report on such a combined indicator, drawing on data that have been collected separately for mothers and newborns.  More research is needed on women’s ability to report accurately on the timing and content of postnatal care visits. The MICS4 module differentiates between care provided after birth either in the facility or by the birth attendant versus postnatal care visits. Research is needed on whether this improves indicator validity | The data collection approach to postnatal care has changed over time in DHS and MICS. Both current DHS and MICS collect information separately for all mothers and newborns, regardless of place of delivery. | Data from the two international survey programs reflects information on pregnancies that occurred two to five years prior to the survey. This reduces the responsiveness of the indicator to immediate programmatic or policy changes on care during the postpartum period |  |
| **Exclusive breastfeeding** | Confers many health and survival benefits to the baby including protection against gastrointestinal infections. Assists in the mother’s recovery after birth and delays the return to fertility | Measures the prevalence of a protective behavior rather than coverage of an intervention to increase the behavior. Measured by excluding consumption of all other food and liquid, so validity is affected by the range of food groups asked in the survey. | The number of food groups included in the DHS survey protocols has expanded, and MICS only asks about liquids rather than specific foods. | Should respond rapidly to program interventions because the indicator assesses infant feeding behaviors in the 24 hours before the survey interview | Straightforward and easy to interpret |
| **Three doses of DTP** | Reduces mortality from diphtheria, tetanus and pertussis. Considered a marker of health system performance because of the three dose requirement | Information is collected by reviewing vaccination records which may have mistakes and errors can occur during transcription of the data from the record. For children without written records, accurate information depends upon parent recall | May be affected by increasing complexity in vaccination schedules. This may result in changes in the order and type of survey questions and introduce additional challenges with ensuring vaccination records are up to date and accurate. | Should respond rapidly to program interventions because most countries have annual estimates, and the indicator captures information about current proportion of children (aged 12-23 months) who received three doses at the time of the survey | Values are easily understandable |
| **Antibiotic treatment for suspected pneumonia** | Pneumonia accounts for an estimated 18% of child deaths. Pneumonia prevention and treatment can reduce these deaths. | The denominator – children with suspected pneumonia in the two weeks preceding the survey – will vary by season and caretaker reporting which depends upon caretaker knowledge of the drugs used to treat the illness. Because of the seasonal variation of the illness, diagnostic confusion could occur with malaria in the absence of rapid diagnostic tests | The denominator has changed over time to exclude cases in which a cough accompanied by rapid or difficult breathing are now excluded if the problem is not chest related, making trend analyses difficult. Seasonal variations in pneumonia mean that tracking trends may be difficult if surveys are carried out at different seasons during the year. | Responsive to programs because the indicator captures information about sick children two weeks prior to the survey. | The low validity of the indicator reduces its use for informing programs |

*Adapted from papers in the Collection on measuring coverage in maternal and child health, and from *Countdown to 2015 and Health Metrics Network (2011) Monitoring maternal, newborn and child health: Understanding key progress indicators. Geneva: WHO*.

Abbreviations:

DHS Demographic Health Surveys

MICS Multiple Indicator Cluster Surveys

WHO World Health Organization
